# Skills acquisition in cavity preparation in conservative dentistry through virtual haptic simulation

**DOI:** 10.3389/fdmed.2025.1698462

**Published:** 2026-02-06

**Authors:** Sebastiana Arroyo-Bote, Catalina Bennasar-Verger, Daniela Paz-Vallejos, Jorge Dominguez-Perez, Pere Riutord-Sbert, Thais Cristina Pereira

**Affiliations:** 1 Universitat de les Illes Balears Grupo ADEMA SALUT, Palma, Spain; 2 Universitat de Barcelona, Barcelona, Spain; 3 Adema Palma Universe School, Palma, Spain

**Keywords:** operative dentistry, preventive dentistry, simulation training, haptic technology, dental education

## Abstract

**Introduction:**

Virtual reality-based training tools have become increasingly incorporated into health education in recent years. In dentistry, three-dimensional (3D) haptic simulators are used in several undergraduate programs to support preclinical training in procedures such as caries removal, cavity preparation, coronal access, prosthodontic preparation, periodontal therapy, and surgical techniques. The aim of this study was to explore student performance in cavity preparation tasks using a virtual haptic simulation system across different academic years of the dentistry degree.

**Methods:**

Second-, third-, and fourth-year dentistry students underwent prior training using 3D haptic simulators in the Virtual Haptic Simulation classroom at ADEMA University School (Universal Simulation, London, UK). Training consisted of completing five cavity preparation activities using preforms, followed by repetition of the same activities with and without the preform. Performance outcomes recorded by the simulator were analyzed and compared between academic years, including precision, surgery time, drilling time, activity progress, target volume, and external volume.

**Results:**

Statistical analysis using ANOVA revealed no statistically significant differences in accuracy among the different academic years (*p* = 0.09915). In contrast, significant differences were observed between groups for surgery time (*p* = 9.059 × 10^−^⁷), drilling time (*p* = 0.0001236), activity progress (*p* = 4.26 × 10^−^⁸), target volume (*p* = 1.244 × 10^−^⁹), and external volume (*p* = 0.005844). No statistically significant differences were identified between cavities prepared with and without the preform in terms of surgery time, drilling time, or activity progress.

**Discussion:**

Within the methodological limitations of this study, including the absence of clinical-transfer assessment, the use of non-equivalent tasks across academic levels, and reliance on simulator-derived metrics with limited sensitivity, the findings should be interpreted with caution. The observed performance patterns reflect students' interaction with the virtual haptic environment rather than definitive evidence of skill acquisition or progressive competence development. Non-significant differences in accuracy between academic years should not be interpreted as equivalence of operative competence. Overall, this study provides descriptive insight into the use of VR-haptic simulation as a supplementary preclinical training resource, highlighting areas for further methodological refinement and future controlled investigations.

## Introduction

Simulation-based medical training tools developed using virtual reality techniques have been increasingly used in health education in recent years (1). This increase occurs not only as a consequence of the COVID-19 pandemic, which caused a decrease in the number of clinical and preclinical practices during quarantine, but also as a way to find an adequate synergy between theoretical knowledge necessary to fully understand clinical procedures and manual best practices. In clinical practices in health education, there are gaps in the number and effectiveness of resources, training and the acquisition of technical skills. The future goal is for medical-surgical institutions to go beyond the minimum standard and adopt new approaches that meaningfully address modern and objective certification of competency (2–6). Practical classes are crucial for the correct training of future health professionals, and virtual simulation can be an efficient educational path to achieve a high level of practice (7).

Virtual techniques provide three-dimensional virtual environments with real-time interaction, which include different degrees of immersion and realism, thus allowing an accurate reproduction of tasks essential for care training procedures (8). Recent studies (9, 10) have highlighted the growing integration of VR-haptic systems in dental education and various dentistry universities use 3D haptic simulators to improve students' practical skills in different treatments like the removal of carious tissues, access cavity, preparations for prostheses, periodontal therapy, surgeries and microsurgeries. These simulators have indirect vision through dental mirrors, exploration probes, high and low speed turbines, surgical drills that simulate real instruments used in clinical practice. Although these instruments are very similar to the real ones, both in shape and in tactile sensitivity, it is necessary to evaluate the extent to which the simulators are effective in improving the learning of dental students in the different dental specialties (11).

There are three types of virtual reality technologies: non-immersive systems use a standard high-resolution desktop system monitor to display the virtual environment and interact with the user through keyboards or other 3D interaction devices; semi-immersive projection systems comprise a relatively high-performance graphics computer system; and fully immersive head-mounted displays provide the most direct experience of virtual environments (12). Haptic systems include tactile information, generally known as force feedback in medical applications (13, 14). VR technology enables three-dimensional computerized images obtained from medical imaging systems to be transformed into patient-specific anatomical models, with added physical properties, providing interaction with the manipulation of realistic models (15–17). The haptic arms simulate the sensation of touch through electric motors in their joints, creating resistance to pressure and differing amounts of torque that simulate the sensation of different hardnesses depending on the tissue being worked on. In addition, the word “haptic” means related to, or coming from, the sense of touch. Haptics, the study of touch, envisions the incorporation of tactile stimuli in virtual reality systems, so the student can experience the sensation of an invasive procedure (18). It is generally accepted that tactile sensation is the most difficult part of an invasive procedure to teach and that improvements in this area of instruction have the potential to speed up the learning curve and improve patient safety. Additionally, a haptic interface is a device that allows a user to interact with a computer through tactile feedback. This feedback is obtained by using a manipulator to apply a degree of force opposed to the user along the x, y, and z axes. Haptic interfaces can be used to simulate operations and actions such as warping and cutting. Three-dimensional haptic devices can be used for applications such as the surgical simulation of complex procedures and training of unskilled surgeons (19).

In this way, the use of haptic simulators together with virtual reality allows students to repeat the procedures an unlimited number of times, shortens the time needed to learn skills, and represents an ecological advance since it limits the waste produced by the use of plastic teeth and other materials in traditional simulation methods. Virtual reality could also eventually be used to certify the clinical skills of professionals as part of continuing professional development (20, 21). However, there are still numerous questions about the use of haptic simulation in dentistry, in particular determining the roles and place to be assigned in dental education programs (22).

Conservative dentistry is considered one of the main specialties of dentistry, since the ability to restore mineralized dental tissues damaged by caries or infectious destructive processes is a basic prerequisite for each and every dentist. The mastery of cavity preparation is taught in every dentistry school through various training strategies meant to address an adequate synergy between the theoretical knowledge needed for a full understanding of clinical procedures and the best manual practices (7). Thus, the use of virtual haptic simulation in the practice of restorative dentistry can be of great help in training future dentists.

Therefore, it is necessary to evaluate whether the use of virtual reality in restorative dentistry enables skills acquisition in cavity preparation. Hence, the objective of this study was to assess the skills acquisition in cavity preparation of students in different years of the dentistry degree. For this, the haptic skills acquisition of dental students in second, third- and fourth-year dentistry were evaluated by carrying out initial familiarization activities, Black Class I and Class II cavities, in virtual simulators, to achieve the minimum pre-established parameters of the different preparations in the virtual simulators.

## Materials and methods

To achieve the designed objectives, 75 dentistry students in the second (*n* = 26), third (*n* = 26) and fourth (*n* = 22) years were taken to the Virtual Haptic Simulation classroom of the ADEMA University School where they were individually positioned in the 3D Haptic Simulators (Universal Simulation, London, UK). Before starting any activity with the simulators, the simulator's haptic arm was calibrated so the students could work in an ergonomic position like real work on dental patients. The haptic arm of the simulators has a simulated turbine that allows students to hold the haptic arm in the same way as they would hold the high-speed dental turbine and the micromotor.

After calibration, students had to choose the activities corresponding to their course: the high-speed turbine option or the H245 31,008 virtual high-speed milling cutter. Each of the activities has blue preforms that determine and highlight the areas of the virtual teeth that need to be removed using the virtual drills. All activities were carried out in the first academic semester. Direct vision mode was used, and the students had no prior exposure to the VR-haptic system beyond institutional orientation. Fourth-year students were engaged in clinical training, although the tasks performed in this study were strictly preclinical within the simulator environment.

First, each student completed five training sessions followed by the evaluated session. In this one, they did the same activities with and without the preform to assess whether they had acquired the skills of virtual cavity preparation.

**Figure 1:**
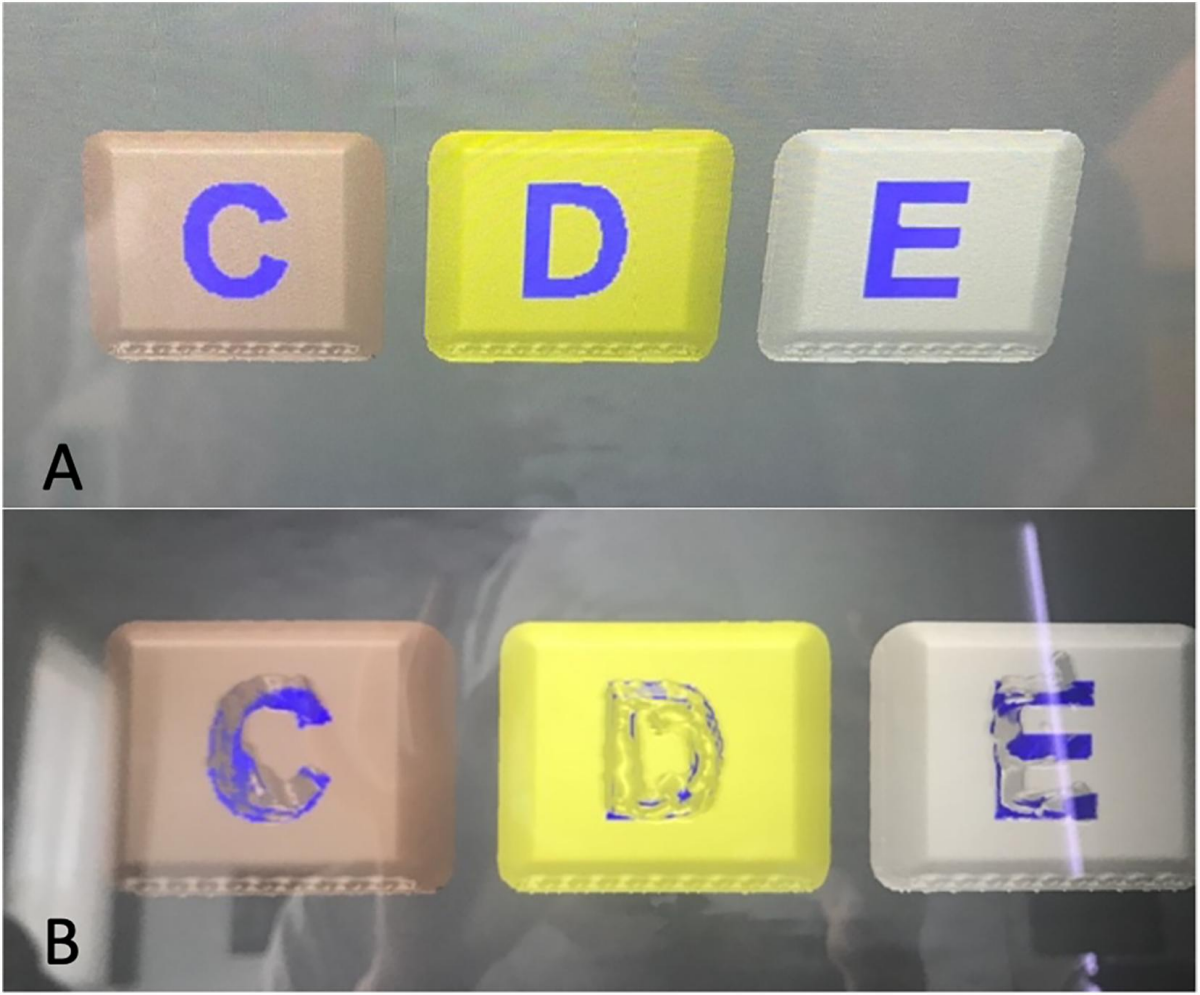
Letters C, D and E of the “Familiarization” part of the virtual simulators that correspond to “Caries”, “Dentin” and “Enamel”, respectively. **(A)** Letters before preparation. **(B)** Letters after preparation by a second-year student.

**Figure 2:**
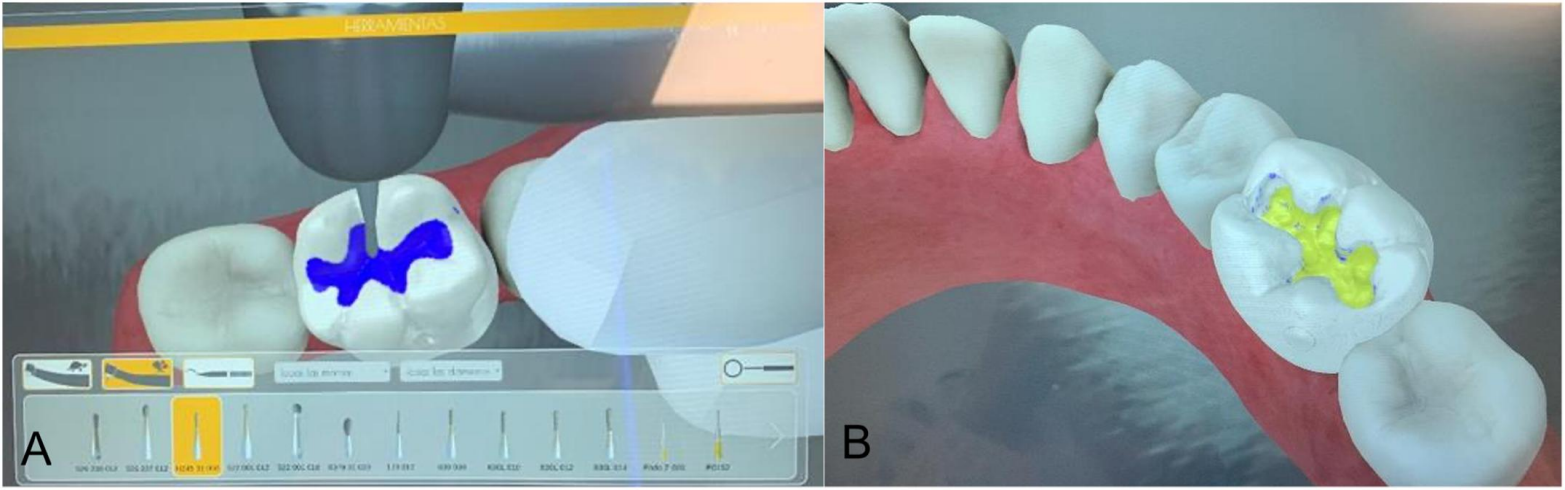
Black class I cavity. **(A)** Before (with preform). **(B)** After cavity preparation.

**Figure 3:**
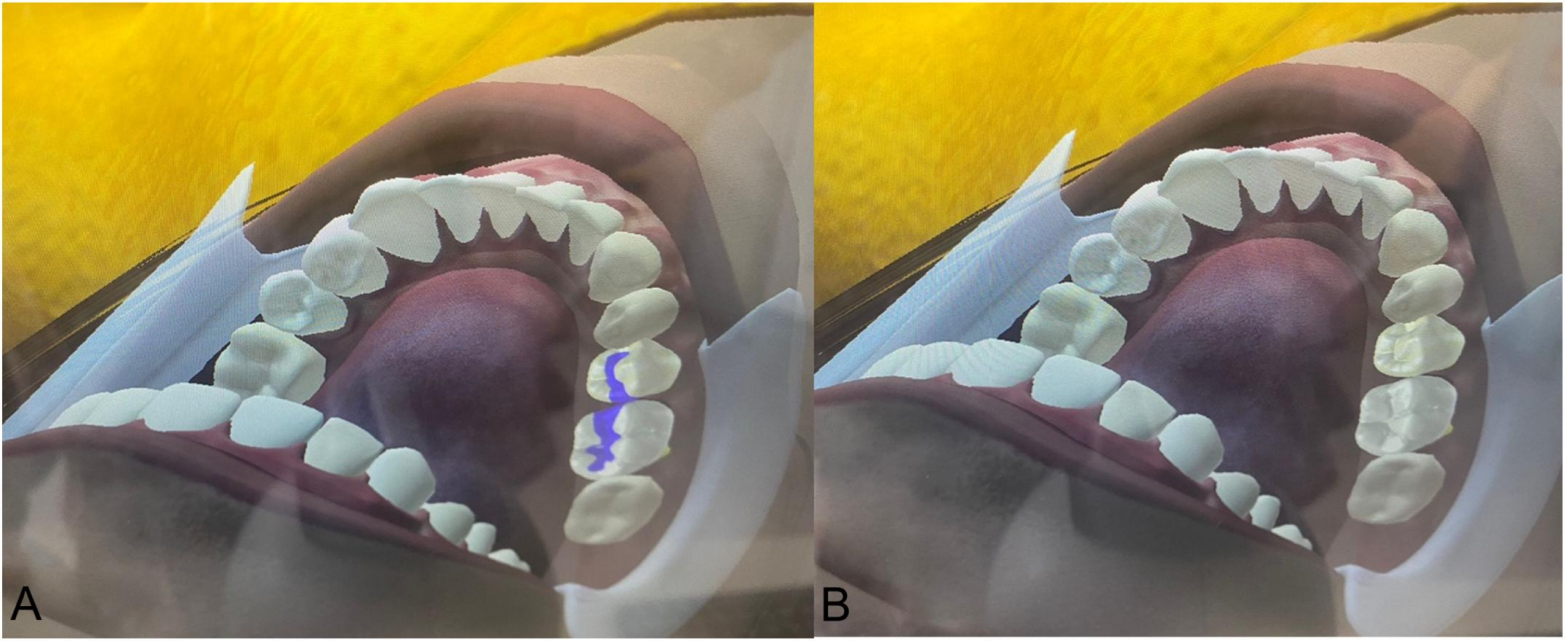
Black class II cavity with **(A)** and without **(B)** preform before cavity preparation.

The students performed the activities according to their skill level acquired in the dentistry degree:

Second-year students (*n* = 26):
  Letters C, D and E of the “Familiarization” part of the virtual simulators, 5 times with preform to practice and once with preform for evaluation. The letters C, D, E, correspond to “Caries”, “Dentin” and “Enamel” respectively and simulate the hardness of each of these tissues (Figure 1).
Third-year students (*n* = 26):
  Black class I cavities in a lower molar in the virtual simulators 5 times with preform and once with and without preform (Figure 2).
Fourth-year students (*n* = 22):
  Black class II cavities in a lower molar in the virtual simulators 5 times with preform and once with and without preform (Figure 3).

The parameters and control values (CV) used were established according to the parameters previously determined in the virtual simulators. These parameters are:

Surgery time: Total time in which the student performed the activity from the moment they touched the tooth for the first time until the activity was finished. The average time established in the simulator was 10 min.
Drilling Time: Total time the student was actively using the drill in the activity. The average time established in the simulator was 8 min.
Progress (PG): Percentage of tissue removed from the total goal of the activity by the student during drilling. Progress means how much “caries” the student has eliminated. A large percentage remaining in the cavity would mean there was “decayed tissue” remaining after the activity ended. The minimum volume of progress established by the simulator was 70%.
Accuracy (PR): Accuracy obtained in the activity, assessing the volume milled by the student that exceeded the limits of the predetermined objective in the simulators (exceeding the preform). The minimum accuracy established for the simulator was 60%.

In addition to these parameters, other results provided by the simulators were also obtained for comparison among students from different years:

Target Volume: Volume in mm^3^ of tissue removed from the target.
External Volume: Volume in mm^3^ of tissue removed outside the target limits.

Once the activities were finished, the students had to save the activities with their username so the results could be accessed through the simulator software, the Virteasy Assistant. Once the results were obtained, the Shapiro–Wilk test was used to contrast normality. For the comparison of the time of surgery, perforation, and progress with the values of the simulators, the Mann–Whitney test was applied, followed by the Wilcoxon test. To compare the results of the students in the parameters Accuracy, Target Volume and External Volume with the parameters of the simulators as well as to compare the abilities among the different years, the ANOVA test was used.

## Results

### Descriptive indications of simulator

**Figure 4:**
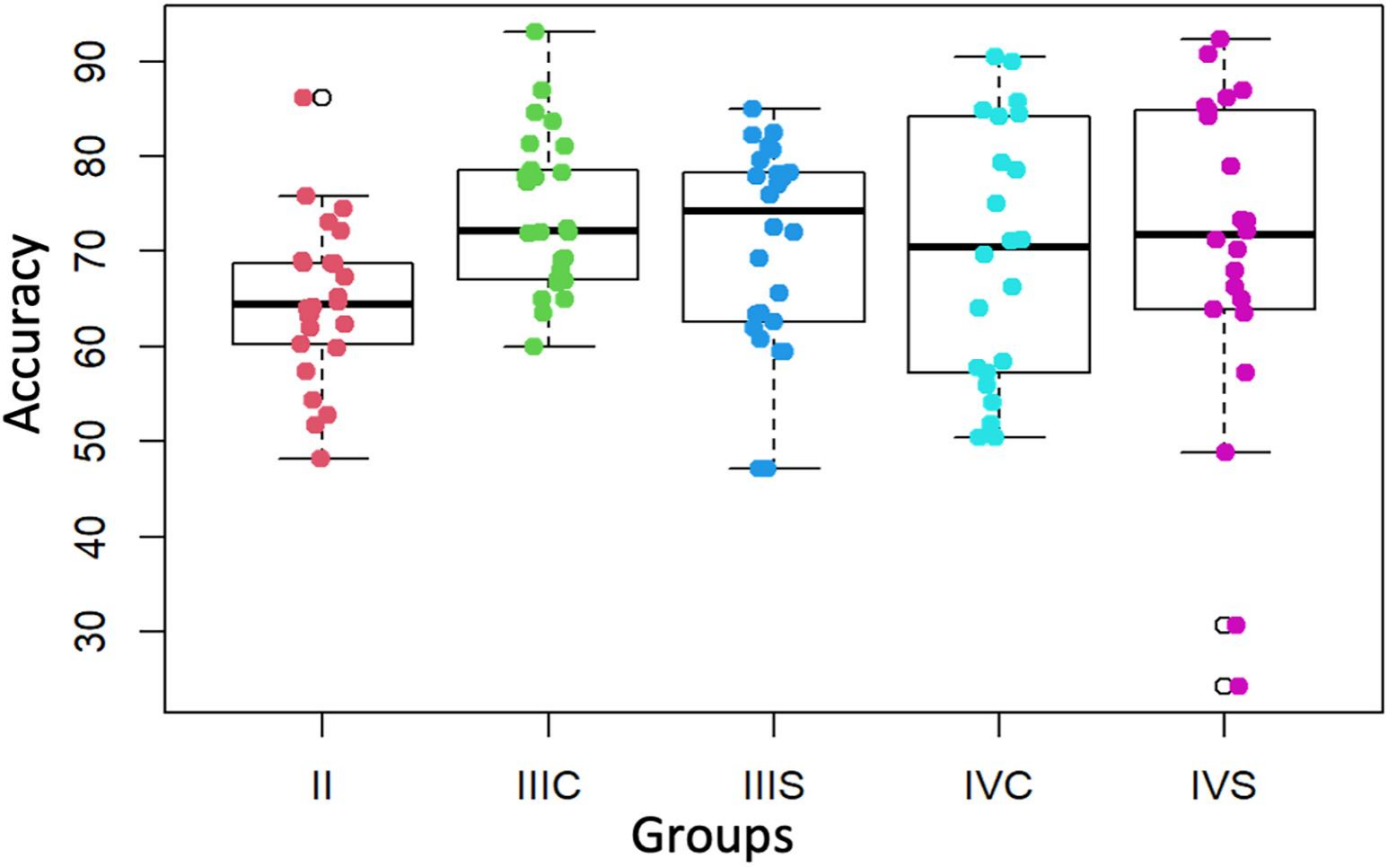
Accuracy values (%) of the second, third and fourth courses (with and without preform). II = second course, IIIS = third course without preform, IIIC = third course with preform, IVS = fourth course without preform, IVC = fourth course with preform.

According to the results of the ANOVA test, the differences observed in the Accuracy values reported in the second, third and fourth year groups are not significant (*p*-value = 0.09915) (Figure 4) in contrast to the differences observed between the different groups in Surgery times (*p*-value = 9.059 × 10^−7^), Perforation times (*p*-value = 0.0001236), Progress (*p*-value = 4.26 × 10^−8^), Target Volume (*p*-value = 1.244 × 10^−9^), and External Volume (*p*-value = 0.005844). The results are described in Tables 1, 2.

**Table 1:** Values of surgery time (minutes), perforation time (minutes), progress (%), accuracy (%), target volume (mm^3^) and external volume (mm^3^).

Variable	II	IIIS	IIIC	IVS	IVC
N	26	26	26	22	22
Surgery time	M = 20.5 IQ = (11, 33)	M = 7.1 IQ = (6.37, 10.6)	M = 9.7 IQ = (7, 13)	M = 17.5 IQ = (10.5, 29)	M = 14.5 IQ = (8.4, 23)
Perforation time	M = 8 IQ = (4.6, 12.6)	M = 3.8 IQ = (3.1, 5.5)	M = 4.8 IQ = (3.3, 6.3)	M = 7.3 IQ = (4.3, 9.6)	M = 5.8 IQ = (4.3, 8.2)
Progress	M = 92 IQ = (88, 93)	M = 98 IQ = (92, 99)	M = 98 IQ = (96, 99)	M = 83 IQ = (52, 98)	M = 57 IQ = (44, 71)
Accuracy	M = 64.4 IQ = (61, 69)	M = 74 IQ = (61, 78)	M = 72 IQ = (67, 78)	M = 72 IQ = (64, 85)	M = 70 IQ = (57, 83)
Target volume	M = 60 IQ = (56, 61)	M = 44 IQ = (43, 44)	M = 44 IQ = (43, 44)	M = 66 IQ = (51, 75)	M = 60 IQ = (45, 77)
External volume	M = 31 IQ = (25, 37)	M = 14 IQ = (12, 26)	M = 13 IQ = (11, 20)	M = 26, IQ = (10, 40)	M = 23 IQ = (9, 43)

II = second course, IIIS = third course without preform, IIIC = third course with preform, IVS = fourth course without preform, IVC = fourth course with preform, M = Median, IQ = interquartile range.

**Table 2:** *P* value of statistical significance of the comparison between the different groups (second, third and fourth years) in relation to surgery time, perforation time, progress, accuracy, target volume and external volume.

Variable	*p*-valor
Surgery time	9.0 × 10^−7^
Perforation time	0.0001236
Progress	4.26 × 10^−8^
Accuracy	0.09915
Target volume	1.244 × 10^−9^
External volume	0.005844

**Figure 5:**
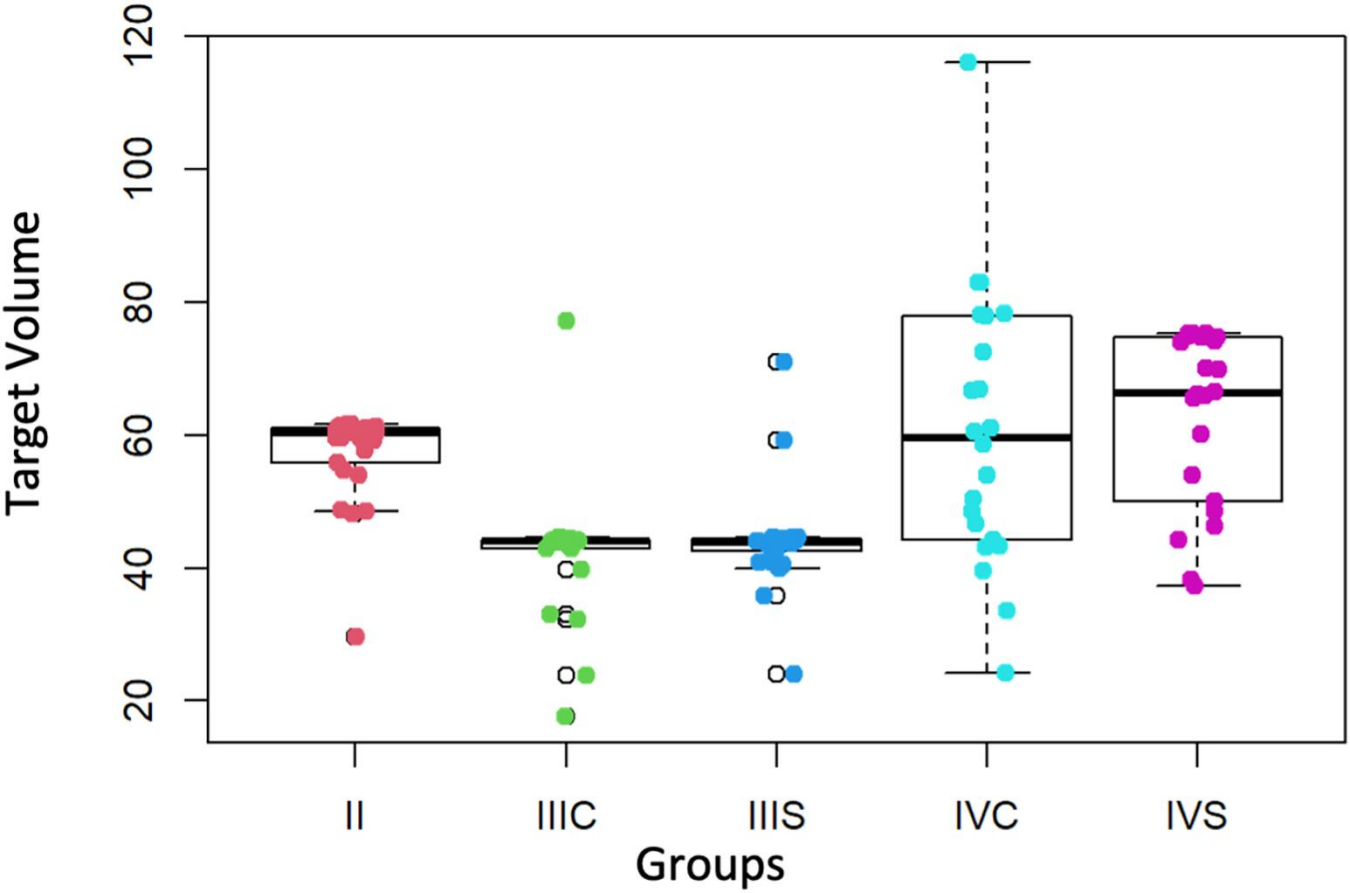
Values of the target volume (mm^3^) of the second, third and fourth courses (with and without preform). II = second course, IIIS = third course without preform, IIIC = third course with preform, IVS = fourth course without preform, IVC = fourth course with preform.

Regarding the “Target Volume” that represents the amount of virtually simulated carious tissue the students would have to remove, the differences observed in the medians of the second year and of the two fourth-year groups (without and with the preform) are not significant (*p*-value = 0.4112), and there is also no difference between the medians of the target volumes of the third year without and with the preform (*p*-value = 0.6786). On the other hand, the median of the target volumes of the third year (without and with the preform) is significantly lower (*p*-value = 8.976 × 10^−12^) than that of the second and fourth year groups (without and with the preform) (Figure 5).

**Figure 6:**
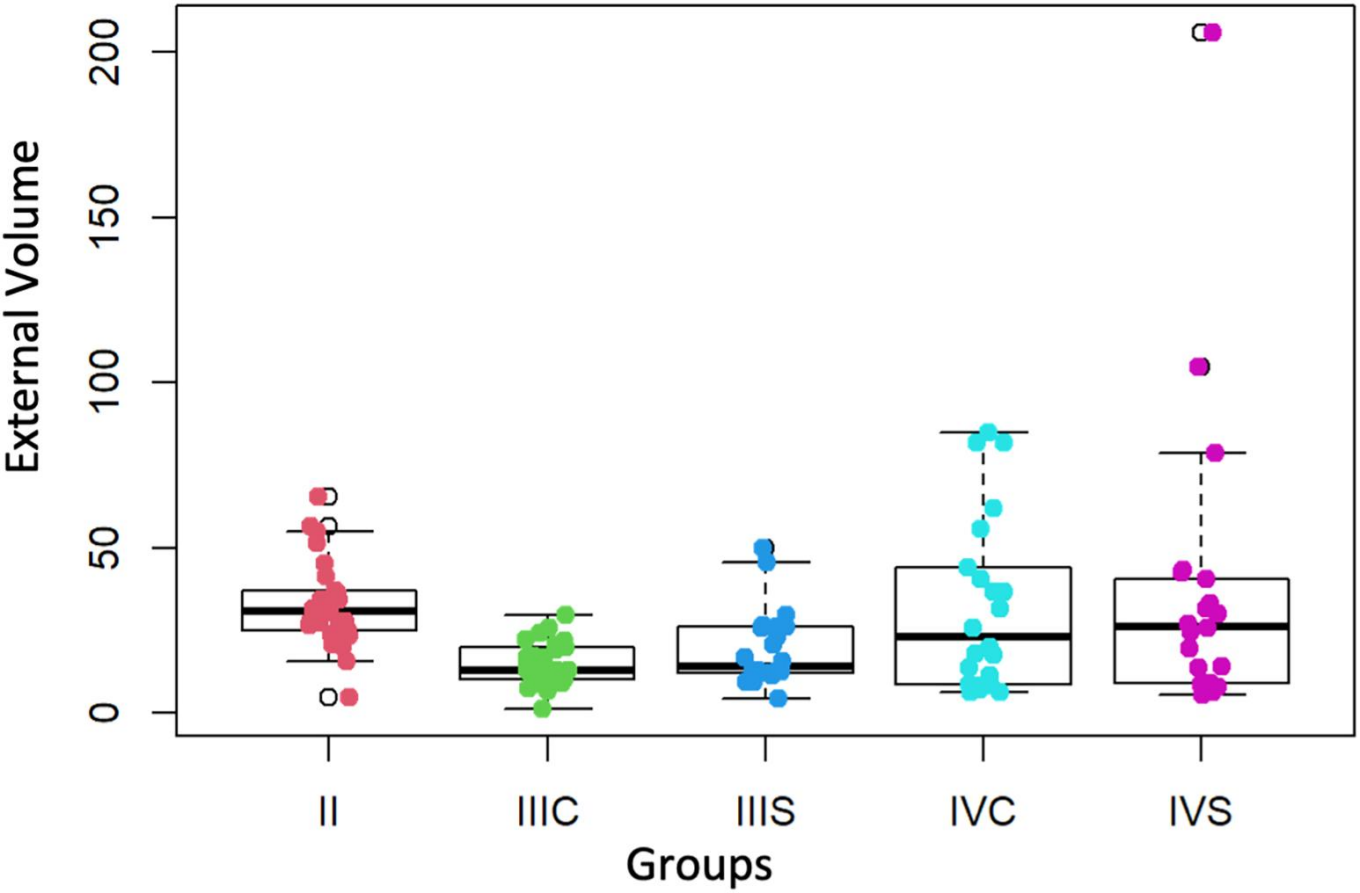
Values of the external volume (mm^3^) of the second, third and fourth courses (with and without preform). II = second course, IIIS = third course without preform, IIIC = third course with preform, IVS = fourth course without preform, IVC = fourth course with preform.

In the evaluation of the External Volume of each year, which means the amount in mm^3^ of simulated “healthy tissue” that was removed, the median corresponding to the fourth year students (25.55 mm^3^) is significantly higher (*p*-value = 0.0266) than that of the third year (12.85 mm^3^). In turn, the median corresponding to the second year (30.8 mm^3^) does not differ significantly (*p*-value = 0.2384) from that of the fourth year (Figure 6).

## Comparison of cavities with and without preform (third and fourth years)

### Surgery time

**Figure 7:**
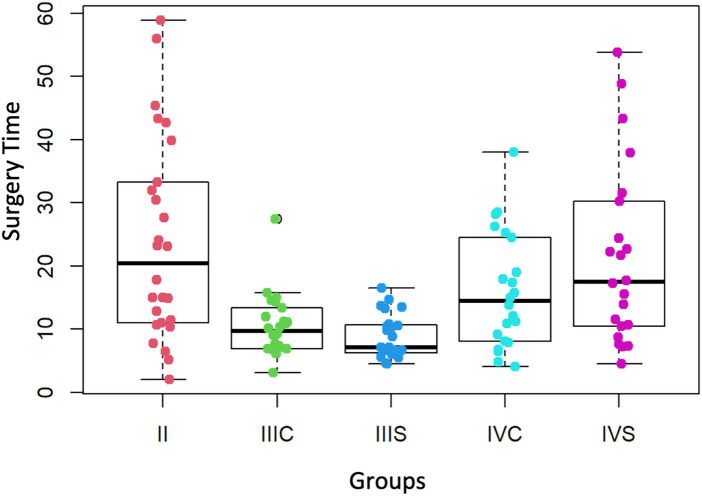
Surgery time (minutes) of the second, third and fourth courses (with and without preform). II = second course, IIIS = third course without preform, IIIC = third course with preform, IVS = fourth course without preform, IVC = fourth course with preform.

The median surgery times corresponding to the third-year group (without preform = 7.1 min and with preform = 9.7 min) (Table 1) are not significantly different from each other (*p*-value = 0.05585). Nor are those corresponding to the fourth-year group (without preform = 17.5 min and with preform = 14.5 min according to Table 1, *p*-value = 0.2348) (Figure 7).

### Drilling time

**Figure 8:**
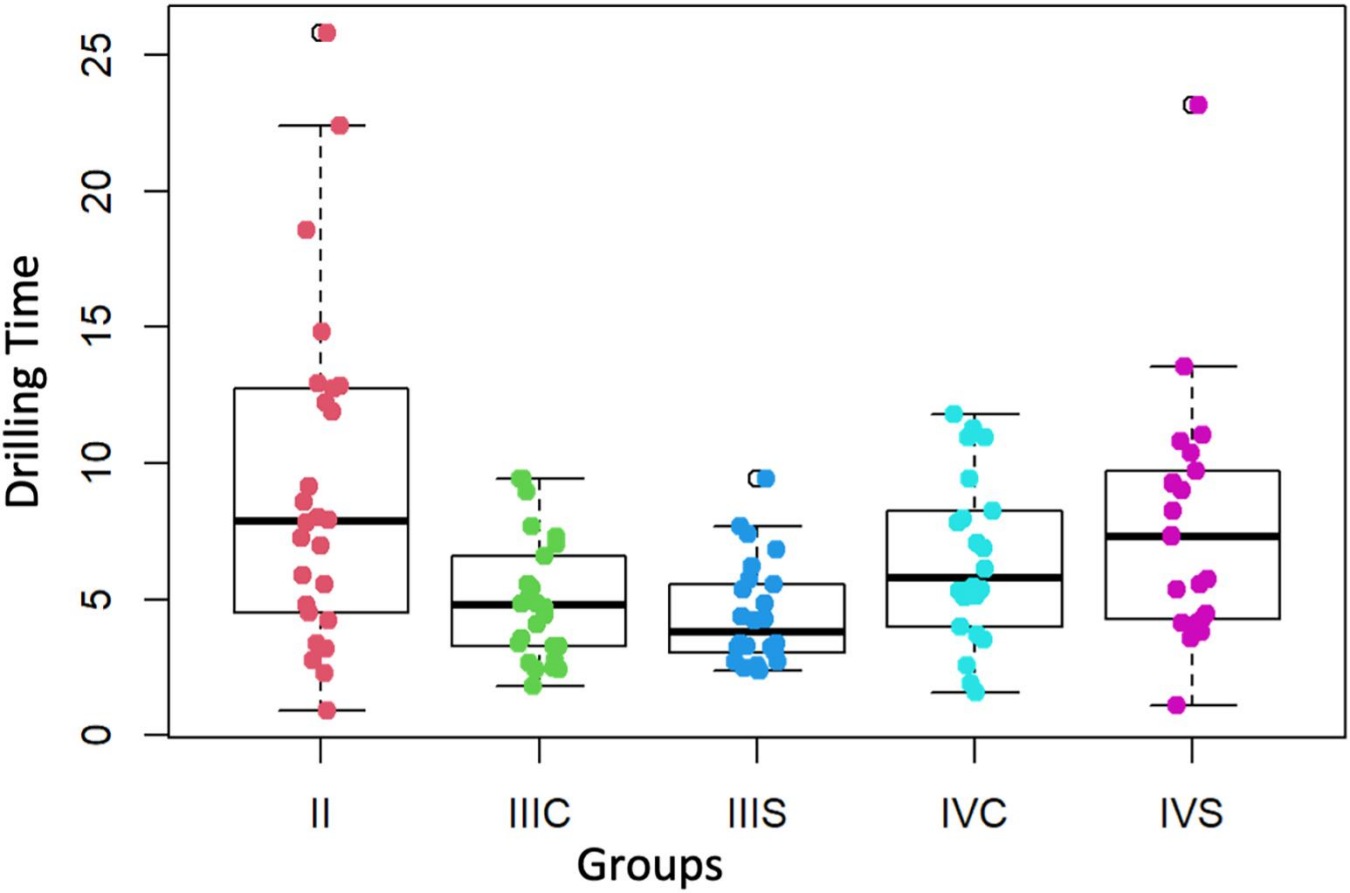
Drilling time (minutes) of the second, third and fourth courses (with and without preform). II = second course, IIIS = third course without preform, IIIC = third course with preform, IVS = fourth course without preform, IVC = fourth course with preform.

The median perforation times corresponding to the third-year group (without preform = 3.8 min and with preform = 4.8 min) (Table 1) are not significantly different from each other (*p*-value = 0.2804). Nor are those corresponding to the fourth-year group (without preform = 7.3 min and with preform = 5.8 min, *p*-value = 0.3535) (Figure 8).

### Progress

**Figure 9:**
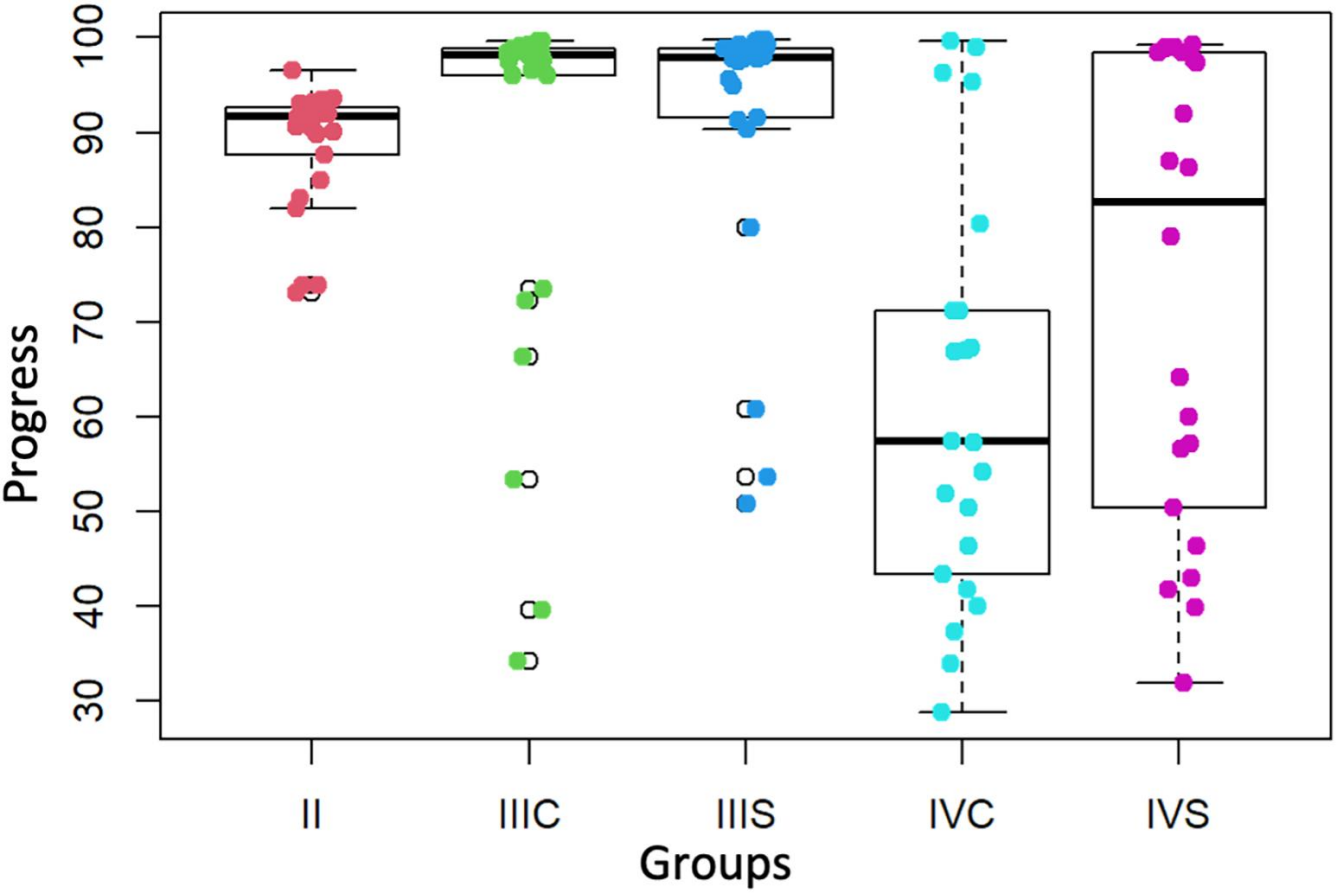
Percentage of progress of the activities of the second, third and fourth years (with and without preform). II = second course, IIIS = third course without preform, IIIC = third course with preform, IVS = fourth course without preform, IVC = fourth course with preform. Comparison of the performance of the students with the parameters of the simulator.

The values of the medians of the progress corresponding to the third year group without and with preform are not significantly different from each other (*p*-value = 0.4512), while the differences between those in the fourth year are significant (*p*-value = 0.03399) (Figure 9).

### Surgery time

The medians of the surgery times corresponding to the second-year group and to the two fourth-year groups (without and with the preform) are significantly greater than 10 min (*p*-values = 9.45×10^−5^, 0.0009413 and 0.01147 respectively). By contrast, the median surgery times of the two groups (without and with the preform) in the third year are not significantly different from the reference value of 10 min (*p*-values = 0.1153 and 0.839, respectively) (Figure 7).

### Drilling time

The medians of the perforation times corresponding to the two groups (without and with the preform) of the third year and to the group with the preform of the fourth year are significantly less than 8 min (*p*-values = 6.9 × 10^−9^, 2.83 × 10^−5^ and 0.0277 respectively). By contrast, those corresponding to the second and fourth years without the preform are not significantly different from the reference value of 8 min (*p*-values = 0.6567 and 0.1903, respectively) (Figure 8).

### Progress

The median progress corresponding to the second year group and the two groups (without and with the preform) of the third year are significantly greater than 70% (*p*-values = 1.49 × 10^−8^, 1.103 × 10^−5^ and 0.001477 respectively). On the other hand, the median progress of the fourth year without the preform does not differ significantly from 70% (*p*-value = 0.1903) while with the preform it is significantly less than 70% (*p*-value = 0.03707) (Figure 9).

## Discussion

The present study sought to evaluate the skills acquisition of students from different years in dentistry in cavity preparation in conservative dentistry through virtual haptic simulation. For this, the students carried out activities with and without a preform, analyzing the surgery time, drilling time, precision, progress, target volume and external volume, which were recorded and compared with the parameters already established in the virtual simulators.

One of the great challenges in teaching dentistry is the clinical training, since this stage requires that students have a diagnostic ability to solve the patient's complaint together with the technical skill to perform the treatments. Due to the complexity of this type of learning, clinical dental treatments performed by a student can result in patient discomfort, risk of complications, and prolonged procedure times (23, 24). For this reason, virtual haptic simulation can be of great help by allowing training and adaptation prior to dental procedures, as they can be repeated as often as desired with no increased cost or risk to the patient (11).

The evaluation carried out in the present study was meant to perform a progressive analysis of the students from their initial stage of contact and adaptation with the dental turbine (second-year students) carrying out familiarization activities up to an intermediate stage in which they must already do Class I Black cavity preparations (third-year students) and the most complex ones involving the proximal tooth surfaces (Black Class II—fourth year students). Therefore, what was evaluated with the second year students was their ability to acquire the competence of sensation and control of the dental turbine, as well as adequate wearing down of the dental structures. Thus, these students did a virtual haptic simulation familiarization activity, which consisted of making preparations in the format of the letters C, D and E that had different densities and hardnesses since they virtually simulated a tissue with caries, dentin and enamel, respectively. In this group, what was really evaluated was their ability to make preparations with different densities, their ability to manually control, to wear down in a straight line or with rotating movements of the turbine; hence, they made the preparations only with the preforms, without going through the phase without the preform. This was the second-year students' first contact with the dental turbine, and having a longer activity explains the significantly higher surgery time values (total time to perform the activity including the moments when the turbine is activated and the moments when it is not) than the parameters of the virtual simulators. It is important to emphasize that the tasks assigned to this group were intentionally introductory and not directly comparable to the cavity preparations performed by third- and fourth-year students. These differences in task type violate ANOVA assumptions, and that statistical comparisons across heterogeneous exercises must be interpreted cautiously.

In the activities of the virtual haptic simulators, there is a goal to be reached, marked in blue, indicating the limits of “tissue” that must be eliminated. The objective is important because from this value it was possible to evaluate the precision with which the students carried out the activities. If they exceeded the limits of the outlined objective, their precision was reduced. In addition, based on the objective, the progress of the activity and the objective volume were evaluated, which shows if the student left “decayed tissue” remaining in the cavity. Progress is a value in percentage and the target volume is in mm^3^. Related to precision, the “external volume” parameter was also evaluated, showing the amount of “healthy tissue” in mm^3^ that was removed. Then, an additional point of interest in our findings is the greater removal of healthy tissue observed among fourth-year students. Several factors may help explain this outcome. More experienced students typically operate with increased speed and confidence, which, although beneficial in clinical settings, may also predispose them to exceeding the ideal preparation boundaries within a virtual environment. Moreover, Class II preparations inherently present greater procedural complexity, requiring more extensive access and instrument angulation, which can increase the risk of over-extension. Motor automatization—developed through repeated clinical practice—may further contribute to less cautious movements when working in a simulated setting. Finally, limitations in the fidelity of haptic feedback may hinder the ability of more experienced students to distinguish fine tactile thresholds, leading to unnecessary removal of sound tissue. This observation underscores the need for continued refinement of simulation metrics and reinforces the importance of training strategies aimed at promoting precision as students advance in their operative skills.

Although a greater removal of healthy tissues for the fourth-year students was found, there were no significant differences in precision across academic levels. While this might initially suggest comparable performance among students with varying degrees of experience, it is more likely attributable to limitations in the precision metrics of the simulator itself. Current VR-haptic systems may lack the resolution necessary to detect subtle variations in fine motor control, particularly when distinguishing between intermediate and advanced users. Recent studies have reported similar challenges, noting that haptic simulators often exhibit ceiling effects or restricted metric sensitivity that constrain their ability to discriminate nuanced performance differences (25, 26). These findings underscore the need for continued technological refinement and the development of more sensitive evaluation parameters to more accurately capture learners' progression in operative precision.

Another important analysis in the present study was the comparison of the ability of third- and fourth-year students to do the same activities without the preform (blue mark) to assess whether they could perform the same activities with the same results. The results showed that there was no statistically significant difference between the cavities made with and without the preform. These results suggest that the previous training the students received with the simulator, using the preforms, trained their manual ability in cavity preparation. These findings agree with the results of the study by Ria et al. (27), who found that beginner dental students had a progressive acquisition of skills and better performance in cavity preparation after practicing with virtual simulators.

All parameters obtained by the virtual haptic simulation allow a precise evaluation of the performance of the students in the activities, which differs from the subjective evaluation of practicing with phantoms or patients (27). Moreover, the primary objective was to assess students’ progression within the VR-haptic environment rather than to compare different training modalities. Nonetheless, a major limitation of this study is the absence of a conventional phantom-head control group, which restricts the generalizability of the findings.

Looking ahead, future research should explore the implementation of adaptive haptic-feedback algorithms capable of dynamically adjusting resistance, tactile cues, and error signals based on the learner's performance. According to recent developments described by Ribeiro et al. (28), such systems can enhance psychomotor calibration by providing individualized feedback pathways that respond to the user's real-time motor patterns. Integrating this type of adaptive technology into dental VR-haptic platforms may help overcome current limitations in metric sensitivity and improve the system's ability to discriminate subtle differences in operative skill. These advancements could ultimately support more personalized training, foster more efficient learning curves, and strengthen the translation of virtual skills to clinical practice.

The present study explored student performance in cavity preparation tasks within a virtual haptic simulation environment across different academic levels. While students were able to complete simulated tasks within the predefined parameters of the system and reproduce cavity designs following prior exposure, these findings should not be interpreted as evidence of skill acquisition, progressive competence development, or equivalence between academic levels. The absence of clinical-transfer assessment, the use of non-equivalent tasks, and the limited discriminative sensitivity of simulator-based metrics preclude any conclusions regarding true operative competence or learning progression. In particular, non-significant differences between student groups should not be interpreted as equivalence of performance.

Within these constraints, virtual haptic simulation may be considered a complementary tool for familiarization with operative procedures and for standardized performance monitoring in preclinical education. Further research incorporating equivalent task design, enhanced assessment sensitivity, and validated clinical-transfer outcomes is required to clarify the educational value and curricular role of VR-haptic simulation in undergraduate dentistry.

## Data Availability

The raw data supporting the conclusions of this article will be made available by the authors, without undue reservation.
